# Diagnostic performance of biparametric versus multiparametric MRI for prostate cancer: a noninferiority, confirmatory observer study

**DOI:** 10.3389/fonc.2026.1808035

**Published:** 2026-06-03

**Authors:** Xingyu Fang, Jialin Li, Wei Lv, Lin Liu, Jing Feng, Li Liu, Feng Pan, Yijun Zhang

**Affiliations:** 1 Department of Radiology, the 305 Hospital of PLA, Beijing, China; 2 Department of Laboratory, The 305 Hospital of PLA, Beijing, China

**Keywords:** biparametric MRI, dynamic contrast-enhanced imaging, multiparametric MRI, PI-RADS, prostate cancer

## Abstract

**Objectives:**

This study aimed to assess the noninferiority of biparametric MRI (bpMRI) compared to multiparametric MRI (mpMRI) using Prostate Imaging Reporting and Data System (PI-RADS) in diagnosing prostate cancer (PCa). Additionally, we compared diagnostic performance of bpMRI and mpMRI between expert and non-expert readers.

**Methods:**

A total of 319 men with abnormal serum prostate-specific antigen (PSA) were included in the study and underwent mpMRI examinations. Two expert readers and two non-expert readers sequentially assessed bpMRI and mpMRI using the PI-RADS scoring system, respectively. The noninferiority of bpMRI versus mpMRI was evaluated using the area under the receiver operating characteristic curve (AUROC) alongside the sensitivity and specificity at a threshold of PI-RADS 3. The diagnostic performance and inter-observer agreement of bpMRI and mpMRI were compared between expert and non-expert readers.

**Results:**

The prevalence of clinically significant PCa (csPCa) was 55.5% (177/319), bpMRI showed noninferior diagnostic performance to mpMRI in AUROC (0.896 [95% CI, 0.879-0.943] vs 0.911 [95% CI, 0.861-0.931]; difference of -1.5% [95% CI, -3.1% to 0.1%], p < 0.001), alongside sensitivity (92.8 [95% CI, 90.1-95.6] vs 93.8 [95% CI, 91.2-96.6]; difference of -1.0% [95% CI, -2.0% to 0.0%], p < 0.001) and specificity (69.4 [95% CI, 63.6-73.9] vs 73.1[95% CI, 67.1-76.9]; difference of -3.5% [95% CI, -6.1% to -1.4%], p < 0.01) at a threshold of PI-RADS 3. In bpMRI interpretation, specificity showed significant differences among readers (P = 0.001); expert readers (32.7%, 209/638) and non-expert readers (37.0%, 236/638) showed significant differences in PI-RADS 3 (P = 0.01) and moderate agreement (0.64 [0.53-0.71]).

**Conclusion:**

The diagnostic performance of bpMRI in detecting csPCa was noninferior to that of mpMRI in a selected high-risk cohort of men with abnormal serum PSA. Limited additional benefits of DCE were observed for non-expert readers. Specialized system training of PI-RADS for non-experts or diagnosis under the supervision of expert radiologists might be better alternatives.

## 1 Introduction

Prostate magnetic resonance imaging (MRI) has become an important tool in the detection of prostate cancer (PCa) for men with elevated serum prostate-specific antigen (PSA) (1–4). To standardize MRI diagnosis of prostate cancer, the Prostate Imaging Reporting and Data System (PI-RADS) was developed and implemented. Its latest version (PI-RADS 2.1) recommends adopting a multiparametric MRI (mpMRI) protocol, including T2-weighted imaging (T2WI) and diffusion-weighted imaging (DWI), supplemented by dynamic contrast-enhanced (DCE) MRI using contrast agent injection (5, 6).

In recent years, non-contrast biparametric MRI (bpMRI) has been increasingly applied. It has been shown to achieve comparable diagnostic accuracy for PCa as mpMRI across multiple studies (7–9). Because DCE plays a subordinate and limited role in PI-RADS scoring, while increasing time and financial costs and potentially harmful to patient health (10–12). However, it could improve readers’ diagnostic confidence and plays a safety net role for certain lesions (12–14).

The primary objective of this study was to assess the noninferiority of bpMRI’s diagnostic accuracy relative to mpMRI in diagnosing clinically significant PCa (csPCa). Additionally, we compared diagnostic performance of bpMRI and mpMRI between expert and non-expert readers.

## 2 Materials and methods

### 2.1 Patients

**Figure 1:**
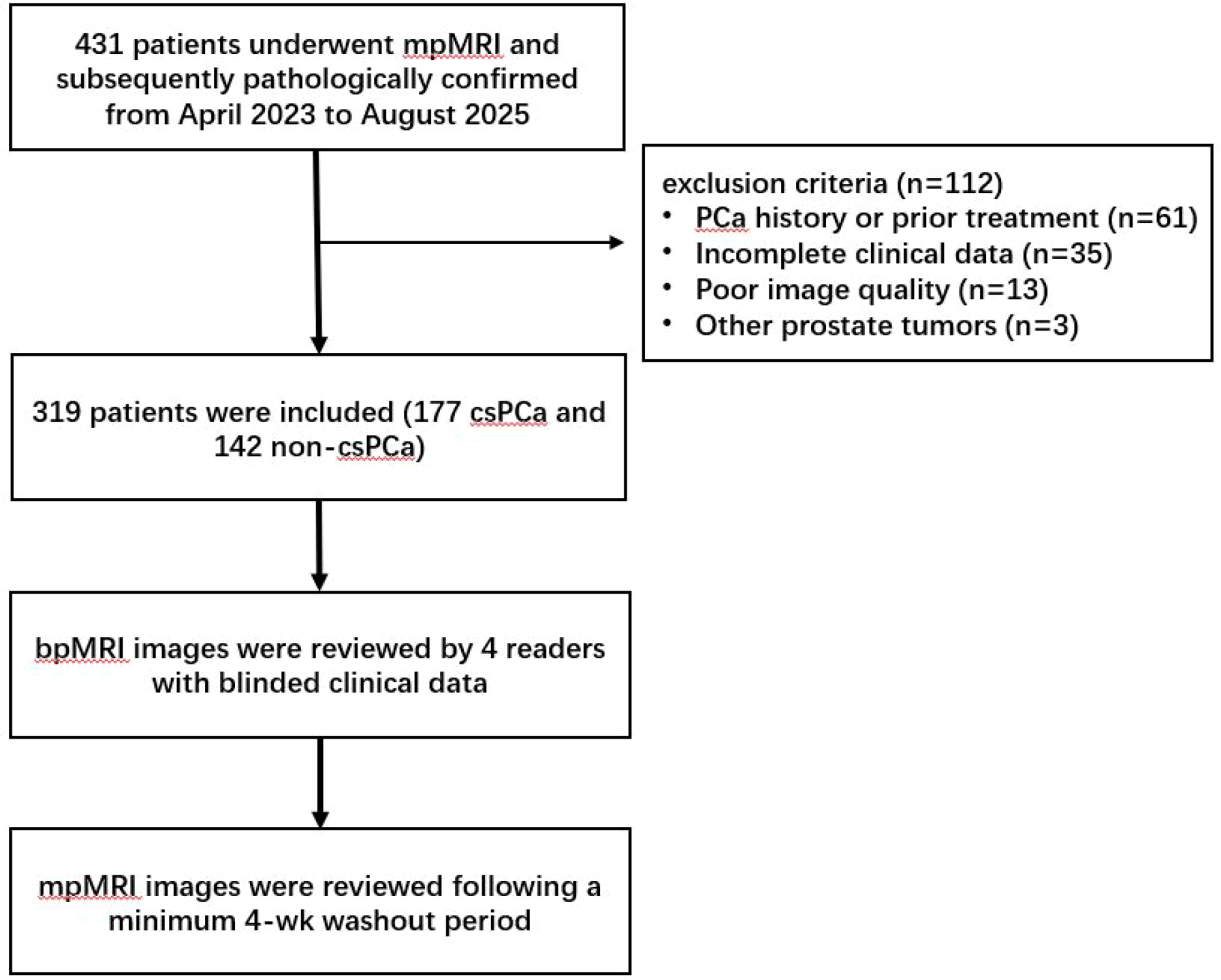
Flowchart of the study participants. mpMRI, multiparametric MRI; bpMRI, biparametric MRI; csPCa, clinically significant prostate cancer.

This study was approved by the Institutional Review Board of the 305 Hospital of PLA. Because the study was retrospective and data were analyzed anonymously, informed consent was exempted. The study was conducted following the principles of the Declaration of Helsinki. A total of 431 consecutive patients were included from April 2023 and August 2025. All examinations were sequentially obtained for a suspicion of PCa, with abnormal serum PSA (≥4 ng/ml) and/or abnormal findings on digital rectal examination. The exclusion criteria included patients with PCa history or prior prostate treatment, incomplete clinical data, poor image quality, other prostate tumors (**Figure 1**). Image quality was assessed through a two-round review process by an expert radiologist (23 years of experience). Severe artifacts such as prostheses, rectal gas, and patient motion were considered as poor image quality.

### 2.2 MRI protocol

MRI examinations were acquired on 1.5 or 3.0 Tesla MRI systems (Signa, GE Healthcare, Chicago, USA; Avanto, Siemens Healthineers, Erlangen, Germany). All MRI protocols were performed in accordance with the requirements of PI-RADS version 2.1. For all examinations, multiplanar (axial, coronal, and sagittal) T2WI, axial T1WI, axial diffused weighted imaging (DWI) and dynamic contrast enhancement were sequentially performed. The apparent diffusion coefficient (ADC) maps were generated from DWI with high b-value of 50, 1000, and 2000 s/mm^2^. The MRI scanning parameters are presented in **Table 1** (15, 16).

**Table 1:** Parameters of sequences of the prostate MRI.

Parameters	FRFSE T2WI Sagittal	FRFSE T2WI coronal	FRFSE T2WI Axial	FSE T1WI Axial	DWI Axial*	DCE
TR, msec	3600	3600	7600	600	5600	4.1
TE, msec	130	130	130	10	80	1.9
ST, mm	3.0	3.0	3.0	3.0	3.0	3.0
FOV, mm	180×180	180×180	180×180	296x220	296x220	296x220
Scan time, min	2:29	2:29	3:12	1:33	4:50	4:30

TR, repetition time; TE, echo time; ST = slice thickness; FOV, field of view; FRFSE, fast recovery fast spin-echo; FSE, fast spin-echo; DWI, diffusion-weighted imaging; DCE, dynamic contrast enhanced imaging.

*DWI performed with b values of 50, 1000, 2000 s/mm^2^.

### 2.3 Image interpretation and measurement

All images were reviewed by two expert radiologists (Z.Y.J, L.L, with 12 and 10 years of experience in reading prostate MRI imaging, respectively) and two non-expert radiologists (L.W, P.F, with 3 and 2 years of experience in reading prostate MRI imaging, respectively). Each radiologist evaluated more than 200 prostate MRI examinations per year. Readers sequentially evaluated bpMRI and mpMRI according to PI-RADS version 2.1. Each reader firstly evaluated bpMRI images for all patients. After a minimum 4-week washout period, readers were allowed to evaluate mpMRI images. The MRI images were sent to the radiologists with all details of the patients’ clinical information blinded. Meanwhile, none of the image interpretations referred to the original radiology reports.

### 2.4 Histopathology and ground truth definition

Every patient underwent both MRI/US fusion-guided biopsy and 12-core systematic biopsy. Before systematic biopsy, MRI/US fusion-guided biopsy was performed and at least 3-core samples were obtained from each patient using a commercial biopsy system (UroNav, Philips, Gainesville, USA). The target lesion was volumetrically delineated on T2WI with DWI and ADC map, then the image-guided biopsy was performed using the MRI/US fusion-guided system. For patients with multiple lesions, at most three lesions were selected for biopsy based on descending PI-RADS scores. Given that all patients present with elevated serum PSA levels, MRI/US fusion-guided biopsies were still performed on the most suspicious areas for patients with PI-RADS 1–2 scores. For patients undergoing radical prostatectomy (RP) following biopsy, the pathological findings obtained after RP were used as the diagnostic gold standard (17). An expert pathologist (20 years of experience) conducted the quality control of the initial pathological results to ensure accuracy and consistency, who was blinded to clinical data and MRI findings. Every specimen was evaluated based on Gleason grade (GG) and Gleason score (GS) according to the International Society of Urological Pathology guidelines (18). The definition of csPCa was GG≥2 (GS = 3 + 4), non-csPCa was defined as GG 1 (GS = 3 + 3). Finally, the data analyst (F.X.) reviewed and compared the quality-controlled pathology results with the PI-RADS scores assigned by each radiologist.

### 2.5 Statistical analysis

The statistical analyses were performed using the SPSS software (version 25, IBM, NY, USA). The cohort was characterized using median with interquartile range (IQR) for non-parametric continuous variables and proportions to describe categorical variables. The non-parametric continuous variables were compared using the Wilcoxon rank-sum test. Comparison of sensitivity and specificity was done using McNemar. Receiver operating characteristic (ROC) analysis was performed and the area under ROC curve (AUROC) was calculated to estimate accuracy. The DeLong method was used to compare the AUROC (19). Noninferiority tests for bpMRI versus mpMRI were conducted with a 5% pre-specified noninferiority margin and significance threshold of p < 0.05, using the AUROC curve as well as the sensitivity and specificity at a threshold of PI-RADS 3 (20). The observer reliability was calculated by the intraclass correlation coefficient (ICC). Absolute agreement, two-way random effects, and single measure models were adopted. The ICC values <0.4, between 0.4 and 0.54, between 0.55 and 0.69, between 0.70 and 0.84, and exceeding 0.85 represented poor, weak, moderate, good, and excellent agreement, respectively (21). Statistical significance was defined as P<0.05.

## 3 Result

### 3.1 Study population

A total of 319 patients were included in the study, and the prevalence of csPCa was 55.5% (177/319). **Table 2** summarize the demographic characteristics of included patients.

**Table 2:** Patient characteristics.

Parameter	csPCa(n=177)	non-csPCa(n=142)	P
Age (year, median [IQR])	65(59-69)	71(65-78)	<0.001
tPSA (ng/ml, median [IQR])	9.3(6.2-11.9)	6.8(4.2-9.8)	<0.001
fPSA (ng/ml, median [IQR])	1.9(0.7-4.1)	1.7(1.1-2.9)	0.08
Prostate volume (cc, median [IQR])	44(34-59)	56(45-69)	<0.001
PSAD (ng/ml/ml, median [IQR])	0.21 (0.16-0.29)	0.12 (0.09-0.17)	<0.001

csPCa, clinically significant prostate cancer; tPSA, total prostate-specific antigen; fPSA, free prostate-specific antigen; PSAD, prostate-specific antigen density; IQR, interquartile range.

### 3.2 Comparison of diagnostic performance

It was observed that bpMRI had noninferior diagnostic performance to mpMRI in AUROC (0.896 [95% CI, 0.879-0.943] vs 0.911 [95% CI, 0.861-0.931]; difference of -1.5% [95% CI, -3.1% to 0.1%], p < 0.001), as well as sensitivity (92.8 [95% CI, 90.1-95.6] vs 93.8 [95% CI, 91.2-96.6]; difference of -1.0% [95% CI, -2.0% to 0.0%], p < 0.001) and specificity (69.4 [95% CI, 63.6-73.9] vs 73.1[95% CI, 67.1-76.9]; difference of -3.5% [95% CI, -6.1% to -1.4%], p < 0.01) at a threshold of PI-RADS 3.

**Figure 2:**
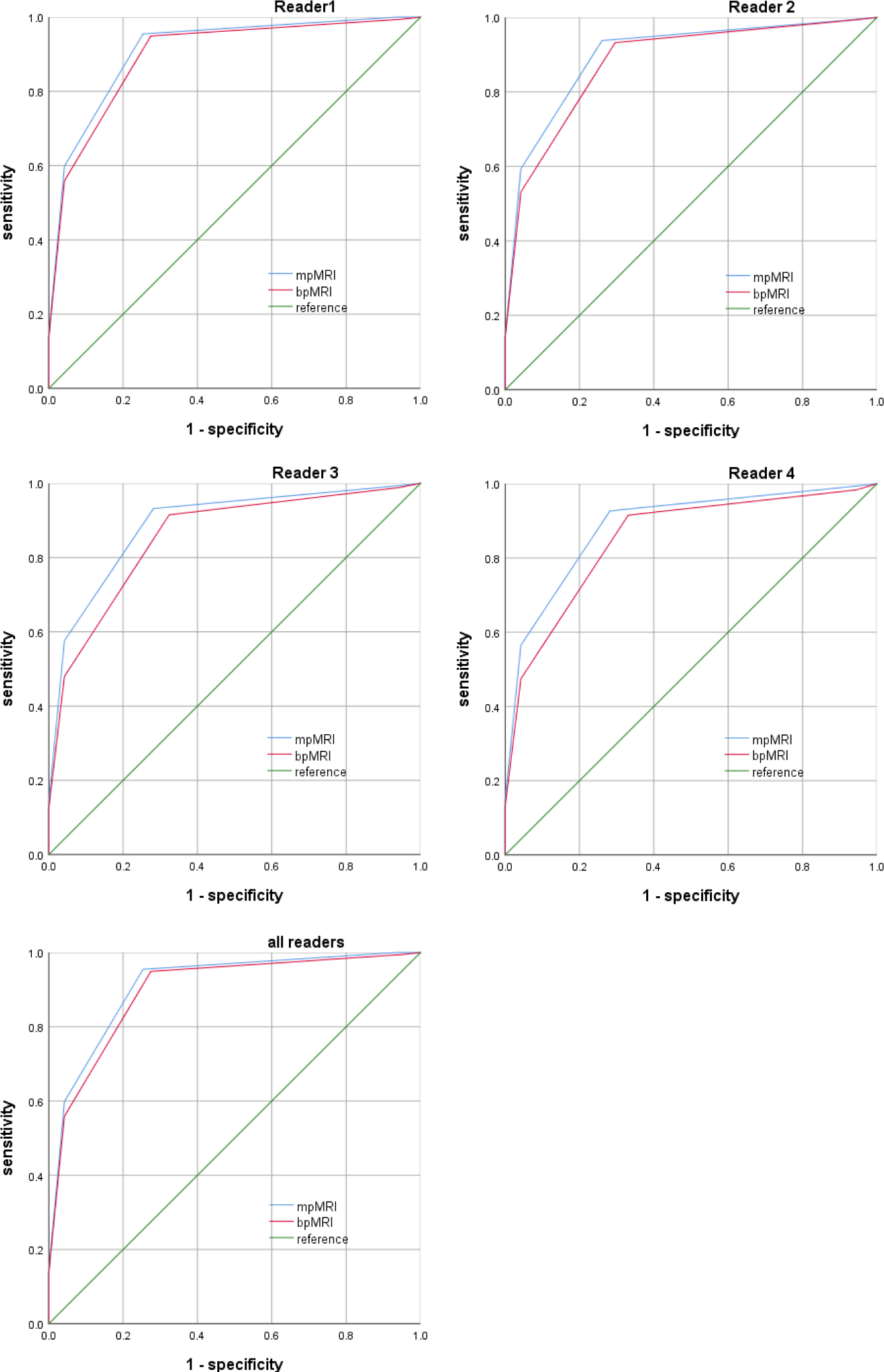
ROC curves for diagnosing clinically significant prostate cancer with bpMRI and mpMRI of four readers. ROC, receiver operating characteristics; mpMRI, multiparametric MRI; bpMRI, biparametric MRI.

For each reader, the AUROC, as well as sensitivity and specificity at a threshold of PI-RADS 3 are shown in **Table 3** and **Figure 2**, also demonstrating that bpMRI was not inferior to mpMRI in diagnostic performance.

**Table 3:** Diagnostic performances and differences of mpMRI and bpMRI.

Parameter		Reader 1	Reader 2	Reader 3	Reader 4	P
mpMRI	Sensitivity % (95% CI)	95.5 (92.1-97.3)	93.8 (89.3-96.2)	93.2 (89.6-95.6)	92.7 (89.2-95.2)	0.26
	Specificity % (95% CI)	74.6 (69.0-79.4)	73.9 (67.9-78.2)	71.8 (65.6-76.1)	71.8 (65.6-76.1)	0.11
bpMRI	Sensitivity % (95% CI)	94.9 (91.5-96.8)	94.4 (88.6-96.7)	91.5 (87.9-93.9)	91.5 (87.9-93.9)	0.13
	Specificity % (95% CI)	72.5 (66.4-76.7)	70.4 (64.6-74.9)	67.6 (62.0-72.8)	66.9 (61.2-72.2)	0.001

bpMRI, biparametric MRI; mpMRI, multiparametric MRI; CI, confidence interval.

### 3.3 Expert versus nonexpert readers

**Figure 3:**
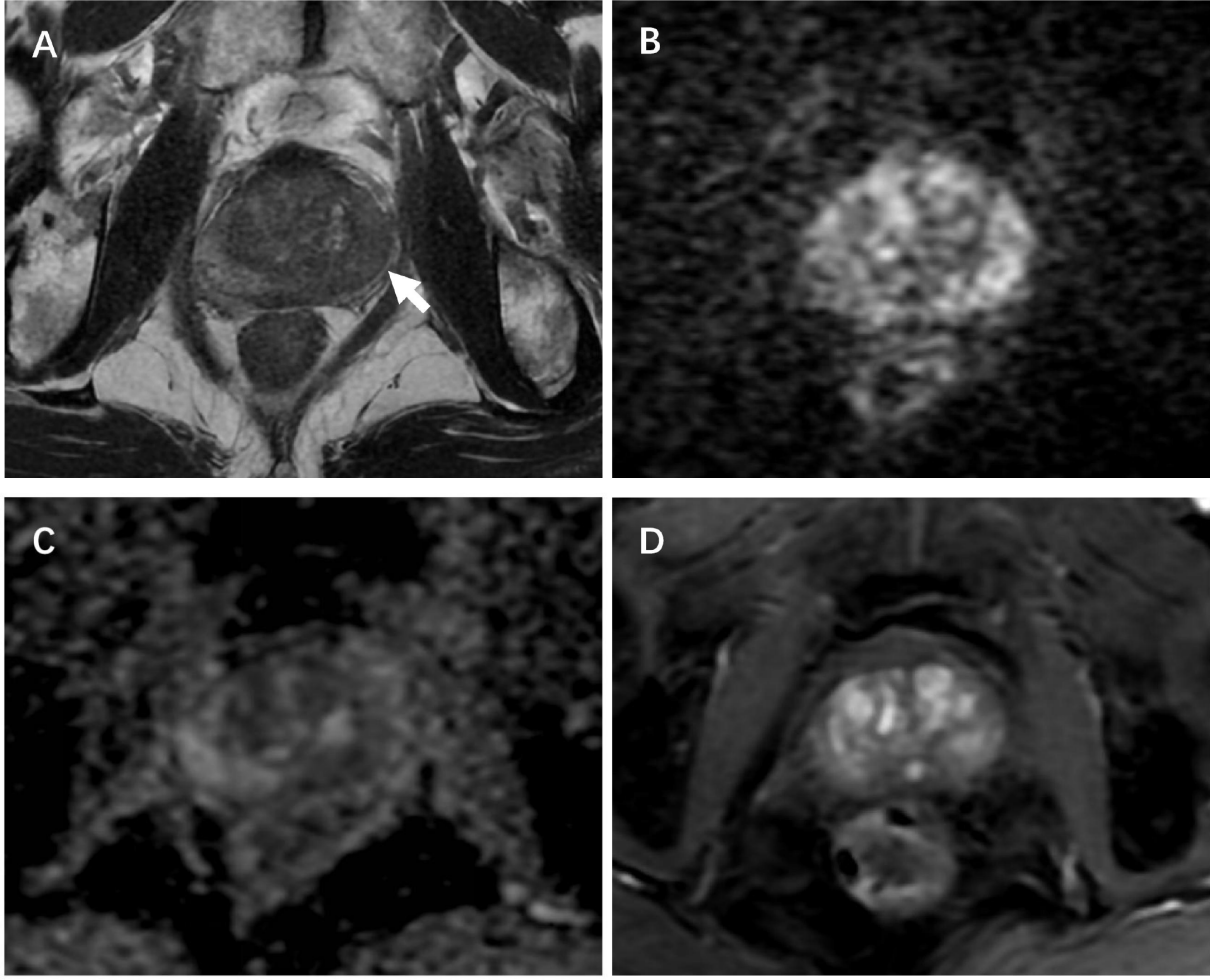
A 68-year-old man with PSA 22.4 ng/ml, were found a lesion (white arrow) in the left peripheral zone on **(a)** axial T2-weighted image, **(b)** axial diffusion-weighted image with b value of 2000 mm2/sec, **(c)** axial apparent diffusion coefficient map, and **(d)** axial dynamic contrast-enhanced image in prostate MRI. The PI-RADS scores assigned by the four readers were 2, 3, 3, and 3 in bpMRI respectively, as well as 2, 2, 3, and 3 in mpMRI respectively. mpMRI, multiparametric MRI; bpMRI, biparametric MRI; PSA, prostate-specific antigen; PI-RADS, Prostate Imaging Reporting and Data System.

In bpMRI interpretation, specificity showed significant differences among readers (P = 0.001). In the non-csPCa group, reader 1 assessed 39 patients (27.5%, 39/142) with PI-RADS 3 or higher, while readers 3 and 4 assessed 46 (32.4%, 46/142) and 47 (33.1%, 47/142) of the patients, respectively (**Figure 3**). Therefore, in the specificity of bpMRI, statistically significant differences were observed between readers 1 and 3, as well as 1 and 4 (both P = 0.001) (**Figure 3**), respectively. There was no difference in the sensitivity and specificity of mpMRI among different readers (both P>0.05).

**Figure 4:**
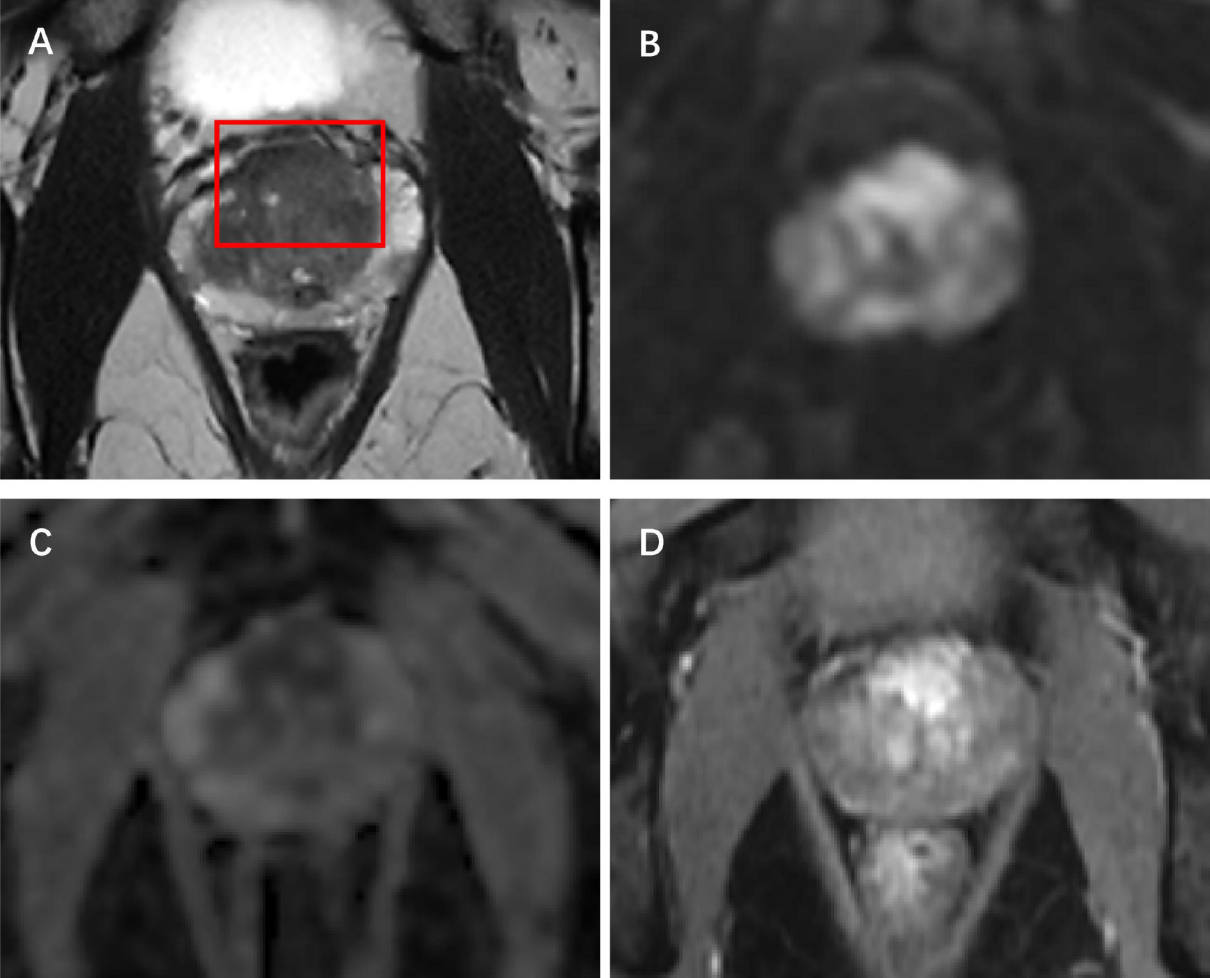
A 66-year-old man with PSA 11.6 ng/ml, prostate MRI revealed a lesion in the transition zone posterior to the anterior fibromuscular stroma (red box) on **(a)** axial T2-weighted image, **(b)** axial diffusion-weighted image with b value of 2000 mm2/sec, **(c)** axial apparent diffusion coefficient map, and **(d)** axial dynamic contrast-enhanced image in prostate MRI. Gleason score 4 + 3 prostate cancer confirmed after radical prostatectomy. The PI-RADS scores assigned by the four readers were 4, 3, 2, and 2 in bpMRI respectively, as well as 4, 4, 2, and 2 in mpMRI respectively. mpMRI, multiparametric MRI; bpMRI, biparametric MRI; PSA, prostate-specific antigen; PI-RADS, Prostate Imaging Reporting and Data System.

In mpMRI interpretation, there was no significant difference between expert and non-expert readers in different PI-RADS scores (all P>0.05). In bpMRI interpretation, expert readers (32.7%, 209/638) and non-expert (37.0%, 236/638) readers showed significant differences in PI-RADS 3 (P = 0.001) (**Figure 4**) (**Table 4**).

**Table 4:** PI-RADS assessment and observer agreement of mpMRI and bpMRI between expert and non-expert readers.

Parameter	mpMRI				bpMRI			
	Expert (n=638)	Non-expert (n=638)	P	Inter-observer	Expert (n=638)	Non-expert (n=638)	P	Inter-observer
PI-RADS 1	18(2.8)	16(2.5)	0.80	0.92 (0.84-0.95)	16(2.5)	19(3.0)	0.59	0.89 (0.80-0.93)
PI-RADS 2	212(33.2)	213(33.4)	0.92	0.82 (0.69-0.88)	208(32.6)	202(31.7)	0.28	0.75 (0.69-0.88)
PI-RADS 3	185(29.0)	195(30.6)	0.27	0.76 (0.63-0.82)	209(32.7)	236(37.0)	0.01	0.64 (0.53-0.71)
PI-RADS 4	173(27.2)	163(25.5)	0.22	0.86 (0.72-0.91)	156(24.5)	136(21.3)	0.07	0.72 (0.67-0.84)
PI-RADS 5	50(7.8)	51(8.0)	0.93	0.99 (0.95-1.00)	49(7.7)	45(7.0)	0.79	0.95 (0.91-0.97)

bpMRI, biparametric MRI; mpMRI, multiparametric MRI; PI-RADS, the Prostate Imaging Reporting and Data System.

The inter-observer ICCs of four readers were 0.86 (0.72-0.91) for mpMRI and 0.84 (0.70-0.89) for bpMRI, respectively, suggesting good to excellent reliability. For PI-RADS scores 1–5 in mpMRI, the inter-observer ICCs between expert and non-expert readers were 0.92 (0.84-0.95), 0.82 (0.69-0.88), 0.76 (0.63-0.82), 0.86 (0.72-0.91) and 0.99 (0.95-1.00), respectively, suggesting good to excellent reliability. For PI-RADS 3 in bpMRI, the inter-observer ICCs between expert and non-expert readers were 0.64 (0.53-0.71), indicating moderate agreement. The other scores sequentially were 0.89 (0.80–0.93), 0.75 (0.69–0.88), 0.72 (0.67–0.84), and 0.95 (0.91–0.97), respectively, suggesting good to excellent reliability.

## 4 Discussion

The use of DCE remained controversial. Studies comparing mpMRI with bpMRI had shown controversial conclusions. A systematic review and meta-analysis integrating 44 studies revealed noninferiority diagnostic performance between mpMRI and bpMRI, with sensitivities of 84% (95% CI, 73-91%) and 89% (95% CI, 80-94%), and specificities of 79% (95% CI, 70-85%) and 74% (95% CI, 56-87%) (7).Other studies had also demonstrated equal performances between bpMRI and mpMRI (8, 9, 22, 23). Whereas, a few papers have reached different conclusions. Tamada et al. (24) found that compared with bpMRI, mpMRI demonstrated higher sensitivity in detecting csPCa, with comparable or lower specificity. Feng et al. (25) demonstrated that the AUROC of mpMRI model was significantly higher than that of bpMRI (DeLong test, P < 0.05). In addition, studies have shown that there was no difference in csPCA detection between different versions of PI-RADS (2.0 versus 2.1) (26, 27). Our study demonstrated that bpMRI was non-inferior to mpMRI in diagnosing csPCa in men without prior history of prostate cancer or treatment, based on AUROC, sensitivity, and specificity at a threshold of PI-RADS 3, consistent with the findings of the majority of studies.

However, for readers with varying levels of experience, the diagnostic performance of bpMRI and mpMRI showed different results. Firstly, statistically significant differences were observed in the specificity of bpMRI among the four readers, whereas no significant differences were found in mpMRI. Notably, non-expert readers overestimated PI-RADS 3 in bpMRI by 4.3% (27/638) compared to expert readers, demonstrating a statistically significant difference between the two groups. In the assessment of PI-RADS 3 in mpMRI, non-expert readers identified only 1.6% (10/638, P = 0.27) more cases than expert readers. For PI-RADS scores of 2 and 4 in bpMRI, non-expert readers assessed 1.1% (5/638, P = 0.28) and 3.2% (20/638, P = 0.09) less than expert readers, respectively. Readers with less experience tend to assign more scores of 3, consistent with previous research (12, 28). Additionally, we found in the assessment of PI-RADS 3 of bpMRI, the inter-observer ICCs between expert and non-expert readers was only moderate 0.64 (0.53-0.71), whereas it was good 0.76 (0.63-0.82) in mpMRI. It indicates that after the absence of DCE’s safenet, non-expert readers were more inclined to make ambiguous diagnoses compared to expert readers, while reducing inter-observer agreement with expert readers.

In previous studies evaluating prostate bpMRI in men with abnormal serum PSA levels, PI-RADS 3 ranged from 10% to 35%, exhibiting a relatively wide distribution interval (29–31). Although the PI-RADS guidelines established standards for prostate MRI reporting, it was well known that interpretation dependent on the reader. One challenge in PI-RADS scoring was that the assessment involved frequent use of qualitative terms rather than quantitative ones, such as mildly, moderately, markedly, especially in DWI and ADC. Moreover, PI-RADS 3 represented an equivocal likelihood of clinically significant prostate cancer, making it moderate diagnosis. Consequently, non-expert readers lacking experience and diagnostic confidence were more likely to assign PI-RADS 3.

Regarding the issues mentioned above in bpMRI, Khalid et al. (32) found that combining PSAD and family history could improve diagnostic performance and reduce the proportion of PI-RADS 3 diagnoses. Additionally, for non-expert application of PI-RADS scoring, an ESUR/ESUI consensus statement recommended specialized training courses, but this approach has not been widely adopted (33).

This study exhibits some strengths. First, every patient underwent both MRI/US fusion-guided biopsy and 12-core systematic biopsy. All patients obtained the maximum extent of pathological evidence, thereby minimizing bias within the overall histopathological cohort. Studies have shown that the use of MRI/US fusion-guided biopsy combined with systematic biopsy, along with appropriate biopsy strategies, could improve csPCA detection (34, 35). Relying on systematic biopsy or follow-up over time to confirm or exclude csPCa has limitations and could be prone to sampling errors (36). Second, we selected multiple readers with varying levels of experience from the same center to demonstrate how experience influences the interpretation of different MR protocols. We believed this design might be more meaningful than a multicenter study, as examination volumes differ across centers. Non-expert readers at centers with higher examination volumes might achieve higher diagnostic accuracy than expert readers at centers with lower volumes.

This study has some limitations. First, this study did not distinguish lesions in different parts of the prostate because its primary objective was to investigate the overall diagnostic performance differences between bpMRI and mpMRI among readers with varying levels of experience. Furthermore, the sample size of the study was relatively small, and conducting subgroup analyses could result in insufficient data. However, PI-RADS applied distinct interpretation criteria for peripheral zone and transition zone, which might result in confounding bias. Second, the sequential rather than cross-over design for reading the bpMRI and mpMRI images have introduced recall or learning bias. Nevertheless, the 4-weeks washout period between two readings significantly minimized this bias, but it could not entirely exclude the possibility that prior exposure to bpMRI influenced mpMRI readings. A randomized crossover design would be preferable in future studies. Third, this study is a single-center retrospective cohort study with a relatively small sample size, which might introduce inherent biases, including selection bias and information bias. Therefore, the findings require further validation through larger-scale, prospective clinical decision studies.

In conclusion, our findings demonstrate that the diagnostic performance of bpMRI in detecting csPCa was noninferior to that of mpMRI in a selected high-risk cohort of men with abnormal serum PSA, as assessed by the AUROC, as well as the sensitivity and specificity at a threshold of PI-RADS 3. However, compared to expert readers in bpMRI interpretation, non-expert readers demonstrated lower specificity in diagnosing csPCa, assigned more PI-RADS 3 diagnoses, and achieved only moderate inter-reader agreement. Therefore, limited additional benefits of DCE were observed for non-expert readers. For centers utilizing bpMRI, professional system training for non-experts or diagnosis under the supervision of expert radiologists might be better alternatives.

## Data Availability

The raw data supporting the conclusions of this article will be made available by the authors, without undue reservation.

## References

[1] Rouvière O Puech P Renard-Penna R Claudon M Roy C Mège-Lechevallier F . Use of prostate systematic and targeted biopsy on the basis of multiparametric MRI in biopsy-naive patients (MRI-FIRST): a prospective, multicentre, paired diagnostic study. Lancet Oncol. (2019) 20:100–9. doi: 10.1016/S1470-2045(18)30569-2 30470502

[2] Drost FH Osses DF Nieboer D Steyerberg EW Bangma CH Roobol MJ . Prostate MRI, with or without MRI-targeted biopsy, and systematic biopsy for detecting prostate cancer. Cochrane Database Syst Rev. (2019) 4:CD012663. doi: 10.1002/14651858.cd012663.pub2. PMID: 31022301 PMC6483565

[3] Cornford P van den Bergh R Briers E Van den Broeck T Brunckhorst O Darraugh J . EAU-EANM-ESTRO-ESUR-ISUP-SIOG guidelines on prostate cancer-2024 update. Part I: Screening, diagnosis, and local treatment with curative intent. Eur Urol. (2024) 86:148–63. doi: 10.1016/j.eururo.2024.03.027. PMID: 38614820

[4] NICE guidance - Prostate cancer: diagnosis and management: © NICE (2019) Prostate cancer: diagnosis and management. BJU Int. (2019) 124:9–26. doi: 10.1111/bju.14809 31206997

[5] Weinreb JC Barentsz JO Choyke PL Cornud F Haider MA Macura KJ . PI-RADS prostate imaging - Reporting and data system: 2015, version 2. Eur Urol. (2016) 69:16–40. doi: 10.1016/j.eururo.2015.08.052. PMID: 26427566 PMC6467207

[6] Turkbey B Rosenkrantz AB Haider MA Padhani AR Villeirs G Macura KJ . Prostate imaging reporting and data system version 2.1: 2019 update of prostate imaging reporting and data system version 2. Eur Urol. (2019) 76:340–51. doi: 10.1016/j.eururo.2019.02.033. PMID: 30898406

[7] Bass EJ Pantovic A Connor M Gabe R Padhani AR Rockall A . A systematic review and meta-analysis of the diagnostic accuracy of biparametric prostate MRI for prostate cancer in men at risk. Prostate Cancer Prostatic Dis. (2021) 24:596–611. doi: 10.1038/s41391-020-00298-w. PMID: 33219368

[8] Alabousi M Salameh JP Gusenbauer K Samoilov L Jafri A Yu H . Biparametric vs multiparametric prostate magnetic resonance imaging for the detection of prostate cancer in treatment-naïve patients: a diagnostic test accuracy systematic review and meta-analysis. BJU Int. (2019) 124:209–20. doi: 10.1111/bju.14759. PMID: 30929292

[9] Twilt JJ Saha A Bosma JS van Ginneken B Bjartell A Padhani AR . Evaluating biparametric versus multiparametric magnetic resonance imaging for diagnosing clinically significant prostate cancer: An international, paired, noninferiority, confirmatory observer study. Eur Urol. (2025) 87:240–50. doi: 10.1016/j.eururo.2024.09.035. PMID: 39438187 PMC11769734

[10] Gulani V Calamante F Shellock FG Kanal E Reeder SB International Society for Magnetic Resonance in Medicine . Gadolinium deposition in the brain: summary of evidence and recommendations. Lancet Neurol. (2017) 16:564–70. doi: 10.1016/s1474-4422(17)30158-8. PMID: 28653648

[11] Carnevale FC Moreira AM de Assis AM Antunes AA de Paula Rodrigues VC Srougi M . Prostatic artery embolization for the treatment of lower urinary tract symptoms due to benign prostatic hyperplasia: 10 years' experience. Radiology. (2020) 296:444–51. doi: 10.1148/radiol.2020191249. PMID: 32484416

[12] Zawaideh JP Sala E Shaida N Koo B Warren AY Carmisciano L . Diagnostic accuracy of biparametric versus multiparametric prostate MRI: assessment of contrast benefit in clinical practice. Eur Radiol. (2020) 30:4039–49. doi: 10.1007/s00330-020-06782-0. PMID: 32166495

[13] Rehman I Pang E Harris AC Chang SD . Bi-parametric prostate MRI with a recall system for contrast enhanced imaging: Improving accessibility while maintaining quality. Eur J Radiol. (2023) 169:111186. doi: 10.1016/j.ejrad.2023.111186. PMID: 37989069

[14] Dell'atti L . Biparametric MRI for local staging of prostate cancer: Current status and future applications. Anticancer Res. (2024) 44:463–70. doi: 10.21873/anticanres.16834 38307562

[15] Yoo JH Kim JY Kim JH . Association between continuous glucose monitoring-derived glycemia risk index and albuminuria in type 2 diabetes. Diabetes Technol Ther. (2023) 25:726–35. doi: 10.1089/dia.2023.0165. PMID: 37335748

[16] Zhang KS Mayer P Glemser PA Tavakoli AA Keymling M Rotkopf LT . Are T2WI PI-RADS sub-scores of transition zone prostate lesions biased by DWI information? A multi-reader, single-center study. Eur J Radiol. (2023) 167:111026. doi: 10.1016/j.ejrad.2023.111026. PMID: 37639843

[17] Montoya Perez I Merisaari H Jambor I Ettala O Taimen P Knaapila J . Detection of prostate cancer using biparametric prostate MRI, radiomics, and kallikreins: A retrospective multicenter study of men with a clinical suspicion of prostate cancer. J Magn Reson Imaging. (2022) 55:465–77. doi: 10.1002/jmri.27811. PMID: 34227169

[18] Epstein JI Egevad L Amin MB Delahunt B Srigley JR Humphrey PA . The 2014 International Society of Urological Pathology (ISUP) consensus conference on Gleason grading of prostatic carcinoma: Definition of grading patterns and proposal for a new grading system. Am J Surg Pathol. (2016) 40:244–52. doi: 10.1097/pas.0000000000000530. PMID: 26492179

[19] DeLong ER DeLong DM Clarke-Pearson DL . Comparing the areas under two or more correlated receiver operating characteristic curves: a nonparametric approach. Biometrics. (1988) 44:837–45. doi: 10.2307/2531595. PMID: 3203132

[20] Chen W Petrick NA Sahiner B . Hypothesis testing in noninferiority and equivalence MRMC ROC studies. Acad Radiol. (2012) 19:1158–65. doi: 10.1016/j.acra.2012.04.011. PMID: 22717591

[21] Schober P Mascha EJ Vetter TR . Statistics from A (agreement) to Z (z score): A guide to interpreting common measures of association, agreement, diagnostic accuracy, effect size, heterogeneity, and reliability in medical research. Anesth Analg. (2021) 133:1633–41. doi: 10.1213/ane.0000000000005773. PMID: 34633993

[22] Knaapila J Jambor I Ettala O Taimen P Verho J Perez IM . Negative predictive value of biparametric prostate magnetic resonance imaging in excluding significant prostate cancer: A pooled data analysis based on clinical data from four prospective, registered studies. Eur Urol Focus. (2021) 7:522–31. doi: 10.1016/j.euf.2020.04.007. PMID: 32418878

[23] Yamaya N Kimura K Ichikawa R Kawanishi M Kawasaki Y Higuchi S . Prospective evaluation of PI-RADSv2.1 using multiparametric and biparametric MRI for detecting clinically significant prostate cancer based on MRI/US fusion-guided biopsy. Jpn J Radiol. (2025) 43:472–82. doi: 10.1007/s11604-024-01675-4. PMID: 39412644 PMC11868320

[24] Tamada T Kido A Yamamoto A Takeuchi M Miyaji Y Moriya T . Comparison of biparametric and multiparametric MRI for clinically significant prostate cancer detection with PI-RADS version 2.1. J Magn Reson Imaging. (2021) 53:283–91. doi: 10.1002/jmri.27283. PMID: 32614123

[25] Feng X Chen X Peng P Zhou H Hong Y Zhu C . Values of multiparametric and biparametric MRI in diagnosing clinically significant prostate cancer: a multivariate analysis. BMC Urol. (2024) 24:40. doi: 10.1186/s12894-024-01411-0. PMID: 38365673 PMC10870467

[26] Ahmet Baytok MK Halil Özer ÖFT Mehmet Kaynar SG Ali FB . Diagnostic performance of PI-RADS V2 and V2.1 and interobserver agreement in both versions. Genel Tıp Derg. (2024) 34:223–9. doi: 10.54005/geneltip.1378687

[27] Tamada T Kido A Takeuchi M Yamamoto A Miyaji Y Kanomata N . Comparison of PI-RADS version 2 and PI-RADS version 2.1 for the detection of transition zone prostate cancer. Eur J Radiol. (2019) 121:108704. doi: 10.1016/j.ejrad.2019.108704. PMID: 31669798

[28] Bosaily AE Frangou E Ahmed HU Emberton M Punwani S Kaplan R . Additional value of dynamic contrast-enhanced sequences in multiparametric prostate magnetic resonance imaging: Data from the PROMIS study. Eur Urol. (2020) 78:503–11. doi: 10.1016/j.eururo.2020.03.002. PMID: 32312543

[29] Boesen L Nørgaard N Løgager V Balslev I Bisbjerg R Thestrup KC . Assessment of the diagnostic accuracy of biparametric magnetic resonance imaging for prostate cancer in biopsy-naive men: The biparametric MRI for detection of prostate cancer (BIDOC) study. JAMA Netw Open. (2018) 1:e180219. doi: 10.1001/jamanetworkopen.2018.0219. PMID: 30646066 PMC6324414

[30] Venderink W van Luijtelaar A van der Leest M Barentsz JO Jenniskens S Sedelaar M . Multiparametric magnetic resonance imaging and follow-up to avoid prostate biopsy in 4259 men. BJU Int. (2019) 124:775–84. doi: 10.1111/bju.14853. PMID: 31237388

[31] Önder Ö Ayva M Yaraşır Y Gürler V Yazıcı MS Akdoğan B . Long-term follow-up results of multiparametric prostate MRI and the prognostic value of PI-RADS: a single-center retrospective cohort study. Diagn Interv Radiol. (2024) 30:139–51. doi: 10.4274/dir.2023.232414 PMC11095067 37724756

[32] Khalid MJ Parker P Smith S Byass OR Cast J . Prevalence of clinically significant prostate carcinoma in Prostate Imaging Reporting and Data System (PIRADS) 3 lesions detected in the peripheral zone on biparametric magnetic resonance imaging (MRI)-a local experience. Clin Radiol. (2024) 79:773–80. doi: 10.1016/j.crad.2024.07.014. PMID: 39129105

[33] de Rooij M Israël B Tummers M Ahmed HU Barrett T Giganti F . ESUR/ESUI consensus statements on multi-parametric MRI for the detection of clinically significant prostate cancer: quality requirements for image acquisition, interpretation and radiologists' training. Eur Radiol. (2020) 30:5404–16. doi: 10.1007/s00330-020-06929-z. PMID: 32424596 PMC7476997

[34] Lee AY Yang XY Lee HJ Law YM Huang HH Sim AS . Limitations of overlapping cores in systematic and MRI-US fusion biopsy. Urol Oncol. (2021) 39:782.e15–782.e21. doi: 10.1016/j.urolonc.2021.02.027. PMID: 33888423

[35] Lee A Chen K Cheng C Ho H Yuen J Ngo NT . Intensive sampling of the umbra and penumbra improves clinically significant prostate cancer detection and reduces risk of grade group upgrading at radical prostatectomy. World J Urol. (2023) 41:2265–71. doi: 10.1007/s00345-023-04499-5. PMID: 37395756

[36] Ziayee F Schimmöller L Boschheidgen M Kasprowski L Al-Monajjed R Quentin M . Benefit of dynamic contrast-enhanced (DCE) imaging for prostate cancer detection depending on readers experience in prostate MRI. Clin Radiol. (2024) 79:e468–74. doi: 10.1016/j.crad.2023.11.026. PMID: 38185579

